# Influence of physicochemical parameters on PPCP occurrences in the wetlands

**DOI:** 10.1007/s10661-022-09990-x

**Published:** 2022-04-07

**Authors:** Chinemerem Ruth Ohoro, Abiodun Olagoke Adeniji, Elsiddig A. E. Elsheikh, Amina Al-Marzouqi, Michael Otim, Omobola Oluranti Okoh, Anthony Ifeanyi Okoh

**Affiliations:** 1 SAMRC Microbial Water Quality Monitoring Centre, University of Fort Hare, Alice, 5700 South Africa; 2 Department of Pure and Applied Chemistry, University of Fort Hare, Alice, 5700 South Africa; 3 Applied and Environmental Microbiology Research Group, Department of Biochemistry and Microbiology, University of Fort Hare, Alice, 5700 South Africa; 4 Department of Chemistry and Chemical Technology, National University of Lesotho. P.O. Roma, 180 Maseru, Lesotho; 5 Present Address: Department of Applied Biology, College of Sciences, University of Sharjah, PO Box 27272 Sharjah, United Arab Emirates; 6 Department of Health Sciences Administration, University of Sharjah, Sharjah, United Arab Emirates; 7 Department of Environmental Health Sciences, College of Health Sciences, University of Sharjah, Sharjah, United Arab Emirates

**Keywords:** Physicochemical properties, Partition coefficient, Pharmaceutical and personal care products, Aquatic environment, Emerging contaminants

## Abstract

There have been many global studies on the occurrence and distribution of pharmaceuticals and personal care products (PPCPs) in the aquatic resources, but reports on the effects of physicochemical properties of water on their concentrations are very scarce. The amounts and removal of these contaminants in various environmental media are dependent on these physicochemical properties, which include pH, temperature, electrical conductivity, salinity, turbidity, and dissolved oxygen. Here, we reviewed the influence of these properties on determination of PPCPs. Reports showed that increase in turbidity, electrical conductivity, and salinity gives increase in concentrations of PPCPs. Also, neutral pH gives higher PPCP concentrations, while decrease in temperature and dissolved oxygen gives low concentration of PPCPs. Nevertheless, it is quite challenging to ascertain the influence of water quality parameters on the PPCP concentration, as other factors like climate change, type of water, source of pollution, persistence, and dilution factor may have great influence on the concentration of PPCPs. Therefore, routine monitoring is suggested as most water quality parameters vary because of effects of climate change.

## Introduction

Organic contaminants such as pharmaceuticals and personal care products (PPCPs) have potentials to cause health hazards and ecological risks (Ohoro et al., 2019; Wang et al., 2022) and find their way to several environmental matrices such as air and dust (Johnson et al., 2011), river water (Anim et al., 2020; Dai et al., 2015), sediments (Ashfaq et al., 2019; Ohoro et al., 2021a, b; Xie et al., 2019), soil, sludge, effluents (Azuma et al., 2019), wastewater treatment plants (Ortiz de García et al., 2014), biota (Mello et al., 2022; Subedi et al., 2012), and clinical samples (Johnson et al., 2011). The concentration of carbamazepine has been detected in environmental samples at alarming rate (Falyouna et al., 2022). The probable risk posed by chemical compounds is dependent on their deadliness and emission state; their physicochemical properties, which manage their distribution, metabolism, and degradation in the environment (Tetko et al., 2013); and their urban land use (Meng et al., 2022). Generally, the assessment of hazard is a very intricate challenge, since chemical compounds, reliant on their physicochemical properties, can influence the environment in very diverse ways. For instance, the effects of some chemical compounds like perfluorinated compounds can last for thousands of years, having a half-life of atmospheric degradation thousands of years. Some are of no concern as they have low solubility (Tetko et al., 2013). Hence, the toxicity effect varies depending on the environmental conditions (Buaisha et al., 2020). Omeprazole, for instance, is the most consumed pharmaceuticals but hardly detected in urban wastewater treatment plants and surface waters. Hence, the metabolites, mainly OTP 5 (omeprazole sulfide), are used in its monitoring program (Boix et al., 2013). Its instability in acid-aqueous solution has been reported (Boix et al., 2013). The degree at which compounds are removed has been proven to be reliant on the physicochemical properties of the compound. The properties of a compound which regulate its fate are aqueous solubility, organic carbon/water partition coefficient (*K*_oc_), octanol/water partition coefficient (*K*_ow_), and Henry’s law constant (*H*_c_) (Langford et al., 2005). Pharmaceuticals’ concentrations in the aquatic systems vary, ranging from the influence of physicochemical properties to tidal factors (Cailleaud et al., 2009; Zhao et al., 2015). To prevent potential factors causing interference with biomarker signals for bio-monitoring research in a fluctuant ecosystem such as estuaries, physicochemical parameters (especially salinity), Cailleaud et al. (2009) suggested that the standardization of sampling processes by opting for a time of the tidal cycle (flow tide and ebb tide) and position in the water column (bottom water or surface, middle column) has to be taken into consideration.

Zhao et al. (2015), in their research, showed that pharmaceutical concentrations decreased with tidal rise and then increased with tidal ebbing for all locations, ascertaining that a particular site which showed an opposite trend was as a result of unusual water movement. The concentrations of pharmaceutical were observed to increase with the concentration of dissolved organic content (DOC), signifying that DOCis a carrier of pharmaceuticals (Zhao et al., 2015). Physicochemical properties of ECs and co-metabolic phenomena seem to affect the biodegradation of compounds (Stasinakis, 2012). Some PPCPs have notable links with physicochemical properties of the water, including pH, dissolved oxygen, chlorophyll *a*, and turbidity (Ferguson et al., 2013; Veach & Bernot, 2011) as well as with the characteristics of the sludge (Stasinakis, 2012). Also, the presence of organic contaminants has been linked to water turbidity (Kerich, 2020). Though total carbon, temperature, total dissolved solids, ammonium ion, and dissolved oxygen were seen as primary factors affecting total pharmaceutical concentrations in water, there was significant disparity in definite factors affecting individual compounds. Such inconsistency is a possible outcome of structural differences among various compounds and intricate biogeochemical interfaces which involves PPCPs. For example, caffeine significantly correlated with nitrate, total carbon, dissolved oxygen, and total dissolved solids; in contrast to a caffeine metabolite, paraxanthine (1, 7-dimethylxanthine), which was negatively affected by pH, temperature, dissolved oxygen, and total dissolved solids (Ferguson et al., 2013). Some of their physicochemical properties are shown in Table 1.

**Table 1:** Partition coefficients of PPCPs

**PPCPs**	**Solubility**^**a**^	***H***_***c***_^***a***^	**pK**_**a**_** (acidity coefficient)**	**Log K**_**ow**_
Diazepam	50	1.5 × 10^−7^	2.92^i^	2.82^j^
Diclofenac	2.4	1.9 × 10^−10^	4.15^e^	4.51^e^
Naproxen	16	1.4 × 10^−8^	4.2^a^	3.18^ l^
Ibuprofen	21	6.1 × 10^−6^	4.9–5.7^a^	33.97^ l^
Sulfamethoxazole	610	2.6 × 10^−11^	5.6^ g^	0.89^f^
Trimethoprim	400	9.8 × 10^−13^	7.3^ h^	0.9–1.4^a^
Erythromycin	1.4	2.2 × 10^−27^	8.9^a^	2.5–3.0^a^
Roxithromycin	0.02	1.0 × 10^−24^	9.2^a^	2.1–2.8^a^
Citalopram	31	1.1 × 10^−9^	9.6^a^	2.9–3.7^a^
Fluoxetine	60	3.6 × 10^−6^	10.1^a^	4.05^a^
Estrone	30	1.6 × 10^−8^	10.4^a^	2.95^n^
17b-Estradiol (E2)	3.6	1.5 × 10^−9^	10.4^a^	3.86^n^
17a-Ethinylestradiol	11.3	3.3 × 10^−10^	10.5–10.7^a^	2.8–4.2^a^
Carbamazepine	17.7	4.4 × 10^−9^	13.9^e^	2.25^ m^
Iopromide	23.8	4.1 × 10^−27^	7.1b	112^ k^
ADBI	0.22	7.3 × 10.1		5.4–6.6a
Tonalide	1.2	4.5 × 10^−3^		4.6–6.4
Galaxolide	1.8	5.1 × 10^−3^		5.9–6.3^a^
Primidone		> 13^b^	0.91^b^	
Tetracycline	3.3, 7.68, 9.69^c^			
Ciprofloxacin	6.43, 8.49^c^	0.28^c^		
Triclosan	7.9^c^	4.76^c^	7.68^ m^	4.66^ m^
Caffeine	10.4^c^		10.4^j^	− 0.07^j^
Bisphenol A			9.73^d^	3.43^d^
N, N-Diethyl-meta-toluamide	0.67^c^	2.18^c^		

^a^Suárez et al. (2008)

^b^Wright (2013)

^c^Yang et al. (2011)

^d^Ozaki et al. (2008), *H*, Henry’s constant

^e^Mello et al. (2022)

^f^Chen and Zhou (2014)

^g^Wu et al. (2013)

^h^Qiao et al. (2011)

^i^Stevens-Garmon et al. (2011)

^j^Rodgers et al. (2005) and Schmitt (2008), Karnjanapiboonwong et al. (2010)

^k^Bonnemain et al. (1990)

^l^Li et al. (2010)

^m^Zhang et al. (2019)

^n^Machatha and Yalkowsky (2005)

This study seeks to (1) evaluate the link between the physicochemical properties of PPCPs and their environmental behaviors, concentrations, and seasonal variation in different environmental media, especially within aquatic systems, and (2) identify possible area of further research. There is paucity of reports on this though. The majority of the documented studies did not report the water quality parameters of the surface waters from where their research samples were collected nor state the correlation of the physicochemical parameters with the concentrations of PPCPs. To the best of our understanding, this is the first article that offers critical evaluation of the link between the physicochemical parameters of waterbodies on the distribution of PPCPs in the environment.

## Effects of physicochemical parameters on PPCPs

### Aqueous solubility, partition coefficient, bioavailability, and transport

The concentration ratio of analyte in octanol to water when the partition between the two phases have reached equilibrium at a specific temperature is known as the octanol–water partition coefficient (K_ow_) (Li et al., 2008). Octanol–air partition coefficient (K_OA_), on the other hand, is known as a fundamental physicochemical property of chemicals partitioning between organic phases and air (Chen et al., 2003).

Adsorption of PPCPs depends on the physicochemical properties (water solubility, vapor pressure, log *K*_ow_) and the half-lives (t_1/2_) in water, air, soil, sediment, etc. (Wang et al., 2015; Yang et al., 2011, 2019), which may have major effects on the uptake and translocation of PPCPs in plants (Yang et al., 2019). The majority of PPCPs show polarity and hydrophilicity with low K_ow_, and there is decrease in concentration due to binding to the organic matter (OM) of sludge or suspended sediments in comparison to other persistent organic pollutants (POPs) that has the capability of bioaccumulation (Yang et al., 2011). The fate and concentration of PPCPs in irrigated soils is related to their physicochemical properties of both sediments and PPCPs which play essential roles in the partitioning of PPCPs between aqueous phase and sediment. Reports on fate and concentration of PPCPs showed that properties of soil/sediment (i.e., free Fe oxides, clay content, pH, OM content, and ion exchange capacity) and properties of compounds (i.e., water solubility, K_ow_, the soil adsorption coefficient (*K*_d_), biodegradation, and acid dissociation constant) have important consequence on the partition of organic contaminants in soil/sediment–water system (Cao et al., 2020; Liu et al., 2020). The OM in sediment can influence the partition of PPCPs principally by hydrophobic interactions. Hydrophobicity of PPCPs restricts the partition of PPCPs, whereas the partition in sediment–water system was less restricted by the molecular weights of PPCPs (Cao et al., 2020). Current studies have shown that PPCPs such as sulfadiazine, sulfamethoxazole, amoxicillin, tetracycline, ciprofloxacin, and trimethoprim can all be adsorbed by plastic fragments (Zhou et al., 2020). The paucity of information on the fate and behavior of the majority of these compounds in the environment has made the pathway of transport of PPCPs difficult to characterize. Also, the pathways of the uptake and bioaccumulation of PPCPs in plants are not clear (Bartrons & Peñuelas, 2017), though it majorly occur via food chain dispersal and aqueous transport (Ebele et al., 2017). Thus, their determination is dependent on their physicochemical properties and persistence and also on the properties of the surrounding matrices (Bartrons & Peñuelas, 2017). Physicochemical properties are vital characteristics impacting the rate at which organic compounds move via soils. Soil type also affects the rate of transport of pharmaceuticals through soils. Soil organic matter (SOM) impedes PPCP migration through soils (Fehsenfeld, 2015). Therefore, more soluble chemicals have a higher potential to seep into groundwater sources, while hydrophobic compounds may be delayed by upper soil layers and be taken up by plants or affect microbial communities (Fehsenfeld, 2015). However, the adsorption to suspended solids or sediment may impact the concentrations of PPCPs in receiving water but not essentially lead to a decrease of their bioavailability or toxicity (Ebele et al., 2017). Occurrence of the low concentration of PPCPs in the effluent of WWTPs might be due to the lack of their degradability by bacteria (Guo et al., 2022) or their low metabolic activity and, also, low bioavailability of PPCPs when their concentration reduced to certain level (Wang & Wang, 2016).

#### Effect of pH

The pH of a particular group of pharmaceuticals was considered by Hao et al. (2006) based on the extraction efficiency of the cartridge or state of the matrix. Based on the latter, Hao et al. (2006) documented that many of the pharmaceuticals under research have both an acidic and a basic functional group that can interact with either the hydrophilic or a lipophilic part of the HLB adsorbent and so eliminating pH interferences during sample extraction. These characteristics can exclude the need for diverse sample pH values to attain the selectivity necessary for sample preparation. The poor recoveries of some pharmaceuticals were attributed to extraction pH (Hao et al., 2006). Similarly, Wu et al. (2008) reported that interferences were co-extracted at pH 3, and less at 5 and 7, which was linked to the factor at neutral condition, that less fulvic and humic acids are co-extracted than in acidic condition (Weigel et al., 2004). Acidic pharmaceuticals apart from ibuprofen were not affected by pH; even the pH variations did not influence neutral compounds (Weigel et al., 2004). Several other studies showed that higher extraction efficiency was obtained at neutral pH, and co-extraction of matrix constituents was considerably reduced at pH 7. Although some compounds like omeprazole had an excellent recovery at pH 8.5 and beta-blockers at pH 10 (Hernando et al., 2007). Extraction efficiency also gave the best recovery at pH 7 than at 2 and 4 (Gómez et al., 2006). Research carried out by Cahill et al. (2004) showed that the low recovery of polar compounds was because the pH of the sample was not adjusted before extraction, thereby giving improper retention on the polymeric sorbent. However, recovery efficiency reduced at the increase of pH from acidic to basic for compounds such as sulfamethoxazole and mebeverine, among others. However, removal rates of pharmaceuticals were higher in acidic media (pH 3–5) (Bui & Choi, 2009). Contrastingly, pH should either be used without adjustment (i.e., 7.5) or at 3 (except a few pharmaceuticals for optimum recovery), during extraction (Grujić et al., 2009).

#### Effect of temperature (^°^C)

Temperature is one of the primary factors influencing total pharmaceutical concentrations, such as caffeine (Ferguson et al., 2013). Preliminary indications suggested that temperature does not play a substantial role in the reduction of the detected compounds in wastewater treatment plants (Lishman et al., 2006). However, the temperature and pressure sufficient to extract the target analytes but at which the interferences remain unremoved from the sewage sludge and surface water samples are necessary to be selected (Göbel et al., 2005; Nieto et al., 2010). In a study conducted in sewage sludge, lower temperature below 100 °C yielded low extraction efficiency. However, a higher temperature above 100 °C decreased the extracted amount as the extract gets darker, indicating the co-extraction of other soluble organic matter and degrading of the expected analytes. Thus, a temperature of 100 °C was chosen as the optimum temperature (Göbel et al., 2005; Nieto et al., 2010). There were cases where the higher temperature was needed to extract specific compounds from antimicrobials, musk fragrances, and some organic compounds utilized in industry, to attain 48–127% recoveries (Nieto et al., 2010). There may be inhibition of microbial growth during summer months, caused by higher temperatures, which may consequently nurture tenacity of pharmaceuticals in aquatic ecosystems. Nevertheless, other in situ researches have reported higher concentrations of pharmaceuticals in freshwaters during winter compared to other seasons of the year (Daneshvar et al., 2010; Veach & Bernot, 2011). Moreover, a complete removal of ibuprofen can be attributed to high temperature in summer as compared with winter season. This suggests that factors such as availability of sunlight, temperature, and precipitation may have a significant effect on pharmaceuticals in surface water as was observed in their transportation and fate in the aquatic environment (Daneshvar et al., 2010). Thus, a report showed consistency with other current studies indicating that concentrations of pharmaceuticals in freshwater are higher in winter months (i.e., December, January, February) with more compounds frequently detected than at other seasons of the year, owing to decreased temperature (Veach & Bernot, 2011; Yuan et al., 2004). However, time of sample collection may give variability of the concentration and persistence of pharmaceutical as was shown by lower pharmaceuticals concentrations in winter caused by lower temperature and fewer overflows (Veach & Bernot, 2011). Meanwhile, some pharmaceuticals such as DEET and sunscreen may have higher concentration in summer than in winter seasons owing to their increased usage in summer (Loraine & Pettigrove, 2006). Furthermore, a report in Wuhan situated in central China, which documented the seasonal variations of hormone contents in STPs, specified that natural estrone and estriol had tendency to have higher concentrations in winter than in summer, possibly as a result of their slow rate of decomposition at low temperature (Liu & Wong, 2013).

Biodegradation of synthetic musks revealed better performance in summer than in fall, which may suggest the influence of temperature and increased solar radiation (Lv et al., 2010).

#### Effect of turbidity (NTU)

The effectiveness of the photochemical transformation procedure in the surface water depends on several environmental factors such as turbidity, the depth of the water column, geographic latitude, weather, shadow (e.g., provided by trees and bushes), and season and abiotic factors such as soil composition, temperature, intensity of sunlight, amount of sunlight, and wavelength of sunlight (here latitude is of importance with regards to temperature), intensity, pH, and salinity (Fatta-Kassinos et al., 2011). Rúa-Gómez and Püttmann, (2012) reported that turbidity of 15 ± 4 NTU had no significant effect on the surface water during sampling of water. Although turbidity restricts sunlight penetration, there is still exposure of water in the top layer and water in secondary clarifiers to sunlight irradiation, particularly in summer. Hence, some contaminants may be influenced by photo-transformation (Fondriest Environmental, 2015; Zhang et al., 2008) and, consequently, decrease dissolved oxygen output (Fondriest Environmental, 2015). Turbidity is generally a result of the dispersion of suspended particles. Abnormal values of turbidity are usually due to resuspension of sediments conveyed by the river inflow from catchment areas. Research conducted by Prasanna and Ranjan (2010) indicated that the turbidity in the estuary is partly influenced by the presence of phytoplankton. High turbidity in the water samples is an indication of pollution and usually due to direct discharge of wastewater into the river (Agedah et al., 2015). PPCPs showed drastic variations in the high turbidity zone (Sun et al., 2016). The lower the salinity, the higher the turbidity in the marine environment and ocean, as the suspended solids combine and settle at the bottom of the river (Fondriest Environmental, 2015). There was a correlation between turbidity with a concentration of PPCPs in the research carried out by Yang et al. (2017). Turbidity increases water temperature because suspended solids absorb heat (Patil et al., 2015).

#### Effect of salinity (PSU)

Salinity can affect the fate and partitioning of organic pollutants as shown by a transparent salinity gradient which was evident when the partition coefficient between water and sediment increases with salinity increase. This is owing to its aqueous solubility decrease which results from the availability of salts (Bowman et al., 2002; Noppe et al., 2007). Results of salinity and partition coefficient published by Bowman et al. (2002) demonstrate that though statistically significant influence on the sorption of 17b-estradiol was not prompted by salinity, it did for estrone. The partition coefficient for both compounds decreased with increasing amount of sediment. Those compounds that show strong salinity dependence are mostly hydrophobic and nonpolar in nature. Estrogens and other steroid hormones are polar in nature, and only few studies on the influence of salinity on the partitioning of polar compounds exist (Bowman et al., 2002). The increase in Kp for estrone that is likely attributed to increased salinity is assumed to be as a result of its decrease in aqueous solubility. This could be linked to the availability of salts, consequential in the more attraction of compound to the particles (salting out) (Bowman et al., 2002; Pal et al., 2010; Wu et al., 2017). Natural waters can have different salt content (e.g., freshwater, seawater), and this will render the studying of fate difficult because of the intricate mixing patterns and effect of salinity as reported by Noppe et al. (2007). The increase in salinity increases the partition coefficient between sediment and water for estrone, resulting in its concentration decrease in extremely saline waters. The extraction efficiency for many pharmaceuticals, including sulfamethoxazole, propranolol, mebeverine, carbamazepine, thioridazine, and diclofenac, was improved with the salinity increase. Nevertheless, the presence of salts instigated a decrease in recovery for monensin but had no major effect on other compounds (Wu et al., 2008; Zhang & Zhou, 2007). Contrarily, the highest concentration of caffeine, metropolol, diclofenac, carbamazepine, sulfonamide, and trimethoprim was obtained at low salinity (Fisch et al., 2021). There was no reasonable difference in the recoveries of the sample with little or no salt content (Hirsch et al., 1998) and with the addition of NaCl (Hilton & Thomas, 2003). Pharmaceuticals are generally hydrophilic compounds with a high water solubility, which will not be considerably influenced by salinity (Zhao et al., 2015). The research reported an apparent change in the form of salinity with the tidal movement. Salinity increased to its maximum value during tidal flow from flood peak to flood slack as a result of continuous seawater coming into the estuary and decreased with freshwater dominating. Consequently, most pharmaceuticals showed a reduction in concentration from flood peak to flood slack in contrast to the salinity response, demonstrating the physical dilution by seawater being an essential procedure monitoring pharmaceutical concentration and then increases in concentration from flood slack to ebb peak and finally to ebb slack (Zhao et al., 2015).

Ramaswamy et al. (2010) attributed the increase of conductivity during the composting process to the effect of the concentration of salts as a result of the degradation of organic matter. The electrical conductivity (EC) has been considered as a marker of pollution by wastewater discharges (de Sousa et al., 2014; Stewart, 2001; Thompson et al., 2012). The measurement of EC by itself is suitable in giving a screening of the level of pollution but when they associate with emerging contaminant concentrations, it can provide definite information about anthropogenic sources of contaminant discharges. EC is certainly a secondary indicator of pollution because it shows a close correlation with the dissolved salt content found in the water column of continental water bodies that frequently is related to sewage discharge and is, consequently, a fixed water quality parameter (de Sousa et al., 2014; Thompson et al., 2012). High EC levels can generally be linked with the occurrence of domestic sewage owing to a rise in the concentration of chloride ion, commonly coming from the human diet, as an average of 6 gof chloride is consumed by each person per day (de Sousa et al., 2014). The EC of 712 ± 50 µs cm^−1^ had no significant effect on the surface water during sampling (Rúa-Gómez & Püttmann, 2012). EC was not correlated with the concentration of PPCPs in the research carried out by Yang et al. (2017). Bisphenol A revealed comparatively low degradation in both brackish and sea water since HCO_3_^−^, an eminent HO^+^scavenger, struggles for accessible HO_+_ in those high conductivity waters (Chu et al., 2017; Gültekin & Ince, 2008). A relationship between salt content and conductivity exists. High electrical conductivity is brought about by increased salt content and high pharmaceutical concentrations (de Sousa et al., 2014).

#### Effect of dissolved oxygen (DO) (mg/L)

Combined sewer overflow charges were observed to decrease the DO levels in the river, introducing pathogenic organisms and causing adverse aesthetic deviations by the discharge of sewage litter, sewage, scum, and grease straight into the river (Munro et al., 2019). Earlier research has revealed that dissolved organic carbon (DOC), fluctuating salinity, and/or suspended particulate matter (SPM) can affect concentrations of drug in tidal waters (Lara-Martín et al., 2014; Munro et al., 2019; Zhao et al., 2015). Consequently, fluctuations in drug concentration were observed over a tidal cycle on a day free from stormwater runoffs or combined sewer overflows to know the influence of fresh/saline water changes (Munro et al., 2019). The high concentration of ammonium, sulfamethoxazole, and ibuprofen was related with low definite conductance, high dissolved oxygen percent saturation, salinity, total dissolved solids, and sites located in the region of study (Ferguson et al., 2013). When there is high organic content in the surface waters, it competes with the dissolved oxygen (Ferguson et al., 2013), thus reducing the DO (Mirra et al., 2020). Work done by de Sousa et al. (2014) showed low compound concentration with high DO concentration. Unambiguously, negative correlations were observed for DO and various individual compounds including salicylic acid, caffeine, acetaminophen, triclosan, naproxen, DEET, and BPA; this may be as a result of the biodegradation increase under aerobic conditions (You et al., 2015). The characteristics and correlation of the physicochemical properties and PPCP concentration are summarized (Table 2).

**Table 2:** Summary of correlation of physicochemical parameter with PPCP concentration

**Physicochemical parameter**	**Units**	**Main characteristics**	**Correlation with the concentration of PPCPs**
Turbidity	NTU	Low turbidity correlates with high salinity	High turbidity will give higher PPCP concentration
pH	pH unit	High pH value reduces the turbidity value	Neutral pH will give higher PPCP concentration
Temperature	^0^C	Turbidity increases water temperature	High temperature will result to low PPCP concentration
Dissolved oxygen	Mg/L	Cold water can hold more dissolved oxygen than warm water	High DO results to low PPCP concentration
Salinity	PSU	The total concentration of dissolved inorganic ions in water	High salinity brings about high PPCP concentration
Electric conductivity	μS/m	Correlates with the total dissolved salt ions in water	High EC brings about high PPCP concentration

It will be challenging to ascertain the undeniable influence of water quality parameters on the contaminants, since other factors such as the water type, seasons, and source of the pollutants may have a great impact on the concentration of the analyte. Secondly, the available reports measured different water quality parameters, making comparison somehow difficult.

From Table 3, it could be inferred that biodegradation of PPCPs occur more often between 19 and 25 °C, indicating that photolysis is the main degradation trail during day time, especially at 22 °C for carbamazepine and triclosan (Yuan et al., 2019). However, high temperature (of about) 45 °C (which was achieved with solar radiation) could aid indirect photolysis of carbamazepine with half-life of 35 h. However, any temperature below the abovementioned values could prevent the growth of bacteria, which would have aided degradation (Yuan et al., 2019). Higher photolysis (photo degradation) takes place in surface water than in wastewater and soil, while higher biodegradation takes place more frequently in wastewater and soil than in surface water. This can be explained using the effect of turbidity as elucidated earlier. Although carbamazepine showed unruly performance with photo degradation in both soil and water, naproxen exhibited higher mobility than triclosan and carbamazepine. Triclosan showed complete degradation both in soil and leachates (Durán-Álvarez et al., 2015). Ibuprofen is the third most consumed drug yet lacks efficiency to biodegrade. For this reason, they are frequently detected in the environment (Chopra & Kumar, 2020). A high concentration (nd to 8.4 µg/L) of ibuprofen had been observed in surface water (Matongo et al., 2015), despite the high temperature of 18–24 °C in agreement to its disorderly behavior to photolysis and biodegradation as stated by Durán-Álvarez et al. (2015), while triclosan at temperature 25.0–25.08 °C gave nd to 0.53 µg/L. Salinity does not play a major role in recoveries of NSAID analysis in water sample as reported by Ngubane et al. (2019). In their study, level of naproxen (0.15–0.36 µg/L) was similar to that reported by Krakkó et al. (2019) (< 0.25 µg/L). They also obtained 0.28 µg/L for ibuprofen in cold season, seemingly at low temperature, compared to nd to 5.3 µg/L reported by Madikizela and Chimuka (2017) at higher temperature. Salinity inhibits hydrolysis of low concentrations of carbamazepine and triclosan (Yuan et al., 2019). Increase in amount of sediment and salinity of the surface water brings about reduction in bioaccumulation of sulfamethoxazole and high concentration in sediment (Chen et al., 2017). From Table 3, the concentration of sulfamethoxazole was low (2.49–24.1 µg/L) when salinity was high, suggesting a possible adsorption in the sediment as reported by Krakkó et al. (2019). Furthermore, the optimal adsorption pH for diclofenac, naproxen, and ibuprofen is 6 (Phasuphan et al., 2019). This could be the reason why the concentration observed by Lindqvist et al. (2005) at pH 5.9–7.0 was higher than the level observed at 7.65–8.36 (Lolić et al., 2015). Triclosan hydrolyzed more in acidic pH than in neutral (Yuan et al., 2019), and degradation of caffeine was found optimum (99.9%) at pH 9.0 (Muthukumar et al., 2020).

**Table 3:** Physicochemical parameters of environmental samples and their respective PPCP concentrations

**Compounds/water quality**	**Water type**	**pH**	**Temp**	**Elec cond**	**Salinity**	**Turbidity**	**DO**	**Conc (**µg/L**)**	**Reference**
Naproxen	Seawater	7.65–8.36	16.1–19.9					nd to 0.178 µg/L	(Lolić et al., 2015)
	River water			331–777	0.16–0.38			nd to 3.8 µg/L	(Madikizela & Chimuka, 2017)
	Raw water	7.30–7.96		0.33–1.10		2.49–24.1		< 0.25 µg/L	(Krakkó et al., 2019)
	Downstream	5.9–7.0	13					0.045 µg/L	(Lindqvist et al., 2005)
	STP	5.9–7.0	13					0.056 µg/L	(Lindqvist et al., 2005)
Ciprofloxacin	Raw water	7.30–7.96		0.33–1.10		2.49–24.1		< 0.025–0.047 µg/L	(Krakkó et al., 2019)
Diclofenac	Raw water	7.30–7.96		0.33–1.10		2.49–24.1		0.033–0.046 µg/L	(Krakkó et al., 2019)
	River water			331–777	0.16–0.38			nd to 5.3 µg/L	(Madikizela & Chimuka, 2017)
	Seawater	7.65–8.36	16.1–19.9					nd to 0.0 241 µg/L	(Lolić et al., 2015)
	Downstream	5.9–7.0	13					0.035 µg/L	(Lindqvist et al., 2005)
	STP	5.9–7.0	13					0.040 µg/L	((Lindqvist et al., 2005)
Tetracycline	Raw water	7.30–7.96		0.33–1.10		2.49–24.1		< 0.025 µg/L	(Krakkó et al., 2019)
Carbamazepine	Raw water	7.30–7.96		0.33–1.10		2.49–24.1		0.023–0.037 µg/L	(Krakkó et al., 2019)
	Surface water	7.08–7.98	25.0–25.80		0.1		2.35 to 8.07	1.35–5.45 µg/L	(Yuan et al., 2019)
	Surface water	7.24–9.20	18–24					2.18–8.896 µg/L	(Matongo et al., 2015)
	Sediment	7.24–9.20	18–24					MDL to 6.07 ng/g	(Matongo et al., 2015)
	Swimming pool	6.86–7.65	26.5–28.9					0.0007–0.0622 µg/L	(Fantuzzi et al., 2018)
Sulfamethoxazole	Raw water	7.30–7.96		0.33–1.10		2.49–24.1		0.014–0.048 µg/L	(Krakkó et al., 2019)
	Surface water	7.24–9.20						nd to 0.24 µg/L	(Matongo et al., 2015)
	Sediment	7.24–9.20						MDL	(Matongo et al., 2015)
Acetaminophen	Sea water	7.65–8.36	16.1–19.9					0.0512–0.0584 µg/L	(Lolić et al., 2015)
	Surface water	7.24–9.20	18–24					0.99–1.74 µg/L	(Matongo et al., 2015)
	Sediment	7.24–9.20	18–24					MDL to 15.8 ng/g	(Matongo et al., 2015)
Ibuprofen	Seawater	7.65–8.36	16.1–19.9					nd to 0.0222 µg/L	(Lolić et al., 2015)
	Downstream	5.9–7.0	13					0.014 µg/L	(Lindqvist et al., 2005)
	STP	5.9–7.0	13					0.011 µg/L	((Lindqvist et al., 2005)
	River water			331–777	0.16–0.38			4.8–11.0 µg/L	(Madikizela & Chimuka, 2017)
	Sediment	7.24–9.20	18–24					MDL to 65.90 ng/g	(Matongo et al., 2015)
	Surface water	7.24–9.20	18–24					nd to 8.4 µg/L	(Matongo et al., 2015)
	Raw water	7.6–7.6	2.2–48.7					< MDL to 0.0333 µg/L	(Reis et al., 2019)
	Swimming pool	6.86–7.65	26.5–28.9					0.0161–0.0197 µg/L	(Fantuzzi et al., 2018)
Ketoprofen	Seawater	7.65–8.36	16.1–19.9					0.0103–0.0897 µg/L	(Lolić et al., 2015)
	Downstream	5.9–7.0	13					0.023 µg/L	(Lindqvist et al., 2005)
	STP	5.9–7.0	13					0.039 µg/L	(Lindqvist et al., 2005)
	Raw water	7.6–7.6	2.2–48.7					< MDL to 0.102 µg/L	(Reis et al., 2019)
	Swimming pool	6.86–7.65	26.5–28.9					0.0007–0.0622 µg/L	(Fantuzzi et al., 2018)
	Swimming pool	6.86–7.65	26.5–28.9					0.0126–0.0127 µg/L	(Fantuzzi et al., 2018)
Bezafibrate	Downstream	5.9–7.0	13					0.004 µg/L	(Lindqvist et al., 2005)
	STP	5.9–7.0	13					0.006 µg/L	(Lindqvist et al., 2005)
Caffeine	Surface water	7.24–9.20	18–24					nd to 3.32 µg/L	(Matongo et al., 2015)
	Sediment	7.24–9.20	18–24					MDL to 1.32 ng/g	(Matongo et al., 2015)
	Raw water	7.6–7.6	2.2–48.7					nd	(Reis et al., 2019)
Erythromycin	Surface water	7.24–9.20	18–24					nd to 5.32 µg/L	(Matongo et al., 2015)
	Sediment	7.24–9.20	18–24					MDL	(Matongo et al., 2015)
	Swimming pool	6.86–7.65	26.5–28.9					0.0024 µg/L	(Fantuzzi et al., 2018)
Paracetamol	Swimming pool	6.86–7.65	26.5–28.9					0.0016–0.0026 µg/L	(Fantuzzi et al., 2018)
Triclosan	Surface	7.08–7.98	25.0–25.8		0.2		2.35 to 8.07	nd to 0.534 µg/L	(Yuan et al., 2019)

*nd* not detected

Furthermore, it is obvious the pH of the matrices from Table 3 ranges between 6.86 and 7.36 for most of the pharmaceuticals. Nevertheless, the concentrations of pharmaceuticals were lower in seawater and swimming pool, downstream, and sewage treatment plant (STP). Other factors could be responsible for this. Firstly, the persistence pharmaceuticals in marine environment is relatively short because of relatively strong photolysis and polarity (Chen et al., 2021). PPCP levels in seawater are expected to show rapid decreases in concentrations when released from sources owing to dilution and diffusion and because of the complex hydrodynamics of the marine environment in coastal zones (Chen et al., 2021; Vidal-Dorsch et al., 2012). Secondly, swimming pools are always kept clean and undergo chlorination treatment, so it is not expected that swimming pool will retain pollutants, since PPCPs are easily degraded by chlorination (Teo et al., 2016); however, some of the non-volatile PPCPs which have slow reaction with chlorine might stay longer in swimming pool (Ekowati et al., 2016). Furthermore, low concentrations were detected in STP probably because there PPCPs occur in low concentrations in STP (Tambosi et al., 2010) or occurrence of possible elimination of the pharmaceuticals from the STP (Vieno et al., 2007).

## Conclusion

The result of the concentration of PPCPs determined in different sample matrices can be affected by their physical and chemical properties and the water quality parameters. However, other factors like climate change and biodegradability of the contaminants may influence the impact of physicochemical parameters on PPCP concentrations; thus, making it quite challenging to determine the occurrence of PPCPs in aquatic environment. Reports from this study showed that increased turbidity, salinity, electrical conductivity, and neutral pH are likely to increase the level of concentrations of PPCPs, while decreased temperature and dissolved oxygen will possibly give decreased PPCPs concentrations. However, studies concerning the effects of physicochemical parameters on PPCP occurrences are still somewhat limited. The following are knowledge gaps and future perspectives to be taken into consideration for further research:

1. Concerning the fate of PPCPs, further research should be carried out using sensitive hyphenated instrument like LC–MS/MS to determine the metabolites of PPCPs especially food products, biota, and water since these matrices are the major route in which these contaminants get into humans and animals.
2. Reports from the reviewed articles did not clearly link the physicochemical parameters to the occurrence of PPCPs nor explain their influence on determination of PPCP concentrations in the environment. Therefore, statistical correlation of PPCP concentration and physicochemical parameters should be included in their research to understand the relationship that exists between them
3. Climate changes influence the occurrence of PPCPs in the environment; unfortunately, there is paucity of reports on this. Therefore, monitoring of PPCPs should be done routinely as most of these physicochemical parameters are unstable because of climate fluctuation.
4. Impacts of physicochemical parameters on PPCP concentrations are scarce in the literature, therefore, research projects designed to assess this area should be conducted. Although the reviewed data showed the influence of some physicochemical parameters on PPCP concentrations, limitation is inevitable: there is lack of sufficient data reviewed. It is essential to explore more studies to validate the claims on this study. However, this study will be essential to direct future research of the occurrence of PPCPs in aquatic environment.
